# PCSK9 inhibitor in acute ischemic stroke patient receiving mechanical thrombectomy: early outcomes and safety

**DOI:** 10.3389/fneur.2024.1375609

**Published:** 2024-05-16

**Authors:** Jonguk Kim, Uichan Hong, Cindy W. Yoon, Jin Woo Bae, Joung-Ho Rha, Hee-Kwon Park

**Affiliations:** ^1^Department of Neurology, Inha University Hospital, Incheon, Republic of Korea; ^2^Department of Neurosurgery, Inha University Hospital, Incheon, Republic of Korea

**Keywords:** mechanical thrombectomy, ischemic stroke, PCSK9 inhibitors, lipid lower medications, functional outcome after acute stroke, hemorrhagic transformation, evolocumab (Repatha)

## Abstract

**Background:**

Lipid-lowering therapies are mainstays in reducing recurrence after acute ischemic stroke (AIS). Evolocumab, a Proprotein convertase subtilisin-kexin type 9 (PCSK9) inhibitor, is a promising lipid-lowering agent known to decrease LDL cholesterol and mitigate vascular events alongside statins. However, its effects on the early functional outcomes post-mechanical thrombectomy (MT) remain unclear. This study aimed to assess the short-term effects and incidence of bleeding events after the early, off-label use of PCSK9 inhibitors in AIS patients undergoing MT.

**Methods:**

We retrospectively analyzed patients who had MT at a Regional Stroke Center from December 2018 to April 2023. Our primary outcome was discharge functional outcomes. Secondary outcomes included early neurologic deterioration (END), symptomatic intracerebral hemorrhage (sICH), 3-month functional outcomes, 3-month recurrence rate, and lipid profiles.

**Results:**

Of 261 patients (mean age 69.2 ± 11.7, men 42.9%), 42 were administered evolocumab peri-procedurally. While baseline characteristics were similar between the two groups, evolocumab group demonstrated improved discharge outcomes, with a lower mean NIHSS (8.8 ± 6.8 vs. 12.4 ± 9.8, *p* = 0.02) and a higher percentage of patients with discharge mRS ≤ 3 (52.4% vs. 35.6%, *p* = 0.041). The 3-month follow-up show a non-significant trend toward an improved outcome in the evolocumab group. Multivariable analysis indicated that evolocumab had a potential impact on favorable discharge outcomes (aOR 1.98[0.94–4.22] for mRS ≤ 3 and 0.47[0.27–0.84] for lower ordinal mRS). Notably, evolocuamb users exhibited fewer instances of END and sICH, although they do not reach statistical significance. Additionally, the evolocumab group demonstrated potential benefits in LDL cholesterol reduction over time.

**Conclusion:**

Early use of evolocumab in AIS patients undergoing MT appeared to be safe and associated with better early functional outcomes. The potential benefit of the PCSK9 inhibitor shown here warrants further prospective studies.

## Introduction

Lipid-lowering therapies, mainly statins, have been shown to reduce the risk of stroke recurrence following an acute ischemic stroke (AIS) and are fundamental to secondary preventive treatment (1, 2). Since gain-of-function mutations in Proprotein convertase subtilisin-kexin type 9 (PCSK9) have been identified as contributors to familial hypercholesterolemia (3, 4), PCSK9 inhibitors have gained attention as novel lipid-lowering treatment. During 2017–2018, two pivotal trials demonstrated that PCSK9 inhibitors, when used in conjunction with high-dose statins, can achieve further reductions in low-density lipoprotein (LDL) cholesterol levels and subsequently decrease the incidence of vascular incidents, including ischemic stroke and myocardial infarction (5, 6).

Beyond the LDL cholesterol regulation, PCSK9 has been shown to have multiple roles in vascular diseases (7–10), PCSK9 inhibitors exhibit anti-inflammatory properties, contribute to the stabilization of atherosclerotic plaques (9), and reduce infarction volume, as seen in *in-vitro* and animal studies (11, 12). These pleiotropic effects of PCSK9 inhibitors have also observed in small human studies (11, 13), with follow-up studies using IVUS showing regression of atherosclerotic plaques (14). Based on these insights, several recent and ongoing studies have been employing PCSK9 inhibitors early in the treatment process and off-label for patients with acute myocardial infarction who are receiving percutaneous coronary intervention (15, 16).

Although the findings from early-phase PCSK9 inhibitor studies in small myocardial infarction cohorts were inconclusive, the potential impact on AIS receiving mechanical thrombectomy (MT) is anticipated, especially considering the direct relationship between final infarction volume and functional outcomes in AIS. A role of PCSK9 inhibitors in the secondary prevention of AIS is relatively well recognized. However, their effectiveness and safety in patients for AIS patients who have undergone MT remain to be explored.

In this study, we retrospectively investigated patients who underwent MT at a regional stroke center to assess whether the early administration of PCSK9 inhibitors is effective in improving the functional outcomes after acute ischemic stroke.

## Methods

### Study population

We retrospectively collected data from patients with acute ischemic stroke who underwent MT at our stroke center between December 2018 and April 2023. Eligibility for inclusion in the study was determined based on the follows: (1) presentation within 24 h after symptom onset, (2) an initial NIH Stroke Scale (NIHSS) score of 2 or higher. Exclusion criteria was as follows: (1) a pre-stroke modified Rankin scale (mRS) score over 2, (2) prior PCSK9 inhibitors treatment before the index stroke, and (3) poor quality of procedure images, or missing data procedure on in-hospital events (a flow chart for patients’ selection presented in Supplementary Figure S1).

### Intervention and treatment

All patients presenting our stroke center with sudden neurologic symptoms received CT or MR angiography with perfusion imaging to determine the presence of large vessel occlusion. When a clinical-core mismatch or core-penumbra mismatch was identified by a neurology specialist, mechanical thrombectomy was performed by one of our five experienced neurointerventionists. A balloon-guide catheter was used as standard practice when applicable.

The choice of thrombectomy technique among contact aspiration, stent retriever, or a combination approach was left to the neurointerventionist’s expertise and judgment. In instances of distal clot migration or clot reformation, pharmacological intervention with intravenous or intra-arterial tirofiban or tissue plasminogen activator (t-PA) was employed. In cases with residual severe stenosis, balloon angioplasty and/or stent insertion were implemented based on the operator’s assessment.

Regarding the administration of evolocumab, a dosage of 140 mg was prescribed prior to the procedure without additional specific indications. Evolocumab was administered subcutaneously, either before the procedure or as promptly as possible afterwards. The prescription of other medications was determined at the discretion of physician according to local guidelines.

### Outcome measure and data collection

The primary outcome of our study was the functional status at discharge, as measured by the mRS. Secondary outcomes included the discharge NIHSS score, in-hospital mortality, the incidence of early neurologic deterioration (END), syptomatic intracerebral hemorrhage (sICH), the 3-month mRS score, and change in the lipid profile (total cholesterol, high-density lipoprotein [HDL] cholesterol, LDL cholesterol, and triglyceride) at the 3-month follow-up.

We systemically collected demographic and clinical data from the prospective registry of our Regional Stroke Center. It included pre-stroke mRS, initial NIHSS score, prevalence of vascular risk factors (hypertension, diabetes, dyslipidemia, atrial fibrillation, prior stroke, current smoking, and obesity [body mass index, BMI ≥ 30 kg/m^2^]), stroke subtypes according to the Trial of Org AST classification (TOAST) (17), prior medications, initial blood pressure, laboratory results (creatinine, C-reactive protein, liver enzymes, Hb A1c), intravenous t-PA use, and discharge medications. We supplemented this data with retrospective chart review when necessary.

Procedure-specific details such as onset-to-door time, door-to-puncture time, puncture-to-recanalization time, the site of occlusion, devices used, the number of devices passes, and the post-procedural reperfusion status [assessed by the modified Thrombolysis in Cerebral Infarction (mTICI)] (18) were collected through the review of intra-procedural images and procedural reports. All procedural images were independently reviewed by two neurointerventionists (J.K. and H-K.P.).

END was defined as any clinical event leading to an increase in the NIHSS score by 2 or more points (19). Symptomatic hemorrhage was characterized as any hemorrhagic transformation on post-procedural CT or MRI that associated with increase of ≥4 points on the NIHSS (20).

The most of baseline characteristic variables we examined had no missing values, although LDL and lipid panels during hospitalization were not recorded in 13.4% and HbA1c in 29.8% of cases. Data on events during hospitalization and outcomes at discharge were collected for all patients; however, 3-month outcomes were missing in 6.5% of cases. Furthermore, data on 3-month lipid panels were only available for 38.3% of cases. These missing values were deleted pair-wisely in the analysis. The data used in this study can be available upon request to the corresponding author.

### Statistical analysis

We compared the baseline characteristics, procedural characteristics, in-hospital events, outcomes at discharge, 3-month clinical outcomes, and 3-month lipid profiles between patients administered evolocumab and those not. We conducted the Chi-square test or Fisher’s exact test for categorical variables and the Student’s t-test or the Mann–Whitney U test for continuous variables. For ordinal mRS score, we employed the Wilcoxon rank-sum test.

We conducted multivariable logistic regression to identify predictors of favorable discharge outcomes, defined as a mRS score of 0–3. Variables that either exhibited a *p*-value ≤ 0.10 in univariate analyses or were considered clinically significant were included. Three models were utilized for this analysis: Model 1 adjusted for demographics and initial stroke severity; Model 2 included variables that were statistically significant in univariate analyses; and Model 3 added variables that differed between evolocumab users and non-users. Additionally, we applied multivariate ordinal regression to mRS scores at discharge and at 3-month, using the same models. All statistical analyses were conducted using the R version 4.3.1.^1^ A *p*-value < 0.05 was considered statistically significant.

### Ethics

The study received a waiver of informed consent from the Inha University Hospital Institutional Review Border (2023-11-040), given the retrospective design and the minimal risk posed to the participants.

## Results

Among 261 eligible patients (mean age 69.2 ± 11.7, men 42.9%), 42 received the evolocumab peri-procedurally without standard indications for PCSK9 inhibitors. Baseline characteristics and comparisons are detailed in Table 1, showing no significant difference in demographics, pre-stroke mRS, most vascular risk factors, stroke subtypes, t-PA use, laboratory findings, and blood pressure between groups. Median initial NIHSS was slightly lower in evolocumab users but was not statistically significant (14 [10.25–18.75] vs. 16 [11–19], *p* = 0.65). Dyslipidemia and atrial fibrillation were less prevalent in evolocumab users, although not statistically significant (19.0% vs. 31.1%, *p* = 0.14, and 33.3% vs. 45.7%, *p* = 0.17, respectively). No significant differences were noted in pre-stroke statin use and discharge statin prescription between groups (14.3% vs. 26.0%, *p* = 0.12, and 92.7% vs. 84.9%, *p* = 0.22, respectively).

**Table 1:** Baseline characteristics of evolocumab user and non-user groups.

	Evolocumab user (*N* = 42)	Non-user (*N* = 219)	Total (*N* = 261)	*p*-value^*^
Male	17 (40.5%)	95 (43.4%)	112 (42.9%)	0.87
Age, mean (SD)	69.8 (12.6)	69.1 (11.6)	69.2 (11.7)	0.72
Pre-stroke mRS				0.86
0	36 (85.7%)	185 (84.5%)	221 (84.7%)	
1	4 (9.5%)	16 (7.3%)	20 (7.7%)	
2	2 (4.8%)	18 (8.2%)	20 (7.7%)	
Initial NIHSS, median (IQR)	14 (10.25, 18.75)	16 (11, 19)	15 (11, 19)	0.65
**Risk factor**				
Hypertension	28 (66.7%)	134 (61.2%)	162 (62.1%)	0.60
Diabetes	11 (26.2%)	63 (28.8%)	74 (28.4%)	0.85
Dyslipidemia	8 (19.0%)	68 (31.1%)	76 (29.1%)	0.14
Atrial fibrillation	14 (33.3%)	100 (45.7%)	114 (43.7%)	0.17
Past stroke history	7 (16.7%)	34 (15.5%)	41 (15.7%)	0.82
Current smoker	14 (33.3%)	56 (25.6%)	70 (26.8%)	0.34
Obesity (BMI ≥ 30)	3 (7.1%)	14 (6.4%)	17 (6.5%)	0.74
Stroke subtype				0.10
Large artery atherosclerosis	16 (39.0%)	53 (24.2%)	70 (26.8%)	
Cardioembolism	14 (34.1%)	100 (45.7%)	114 (43.7%)	
Others	11 (26.8%)	66 (30.1%)	77 (29.5%)	
**Medication**				
IV t-PA use	13 (31.0%)	77 (35.2%)	90 (34.5%)	0.72
Prior statin use	6 (14.3%)	57 (26.0%)	63 (24.1%)	0.12
Discharge Statin prescription^†^	38 (92.7%)	163 (84.9%)	201 (86.3%)	0.22
Discharge antiplatelet^†^	28 (68.3%)	106 (55.2%)	134 (57.5%)	0.16
Discharge anticoagulant^†^	13 (31.7%)	83 (43.2%)	96 (41.2%)	0.22
**Laboratory findings**				
SBP	152.3 (28.6)	152.8 (28.6)	152.8 (28.6)	0.91
DBP	89.3 (15.3)	86.9 (20.0)	87.3 (19.3)	0.46
Creatinine	0.87 (0.32)	0.99 (0.66)	0.97 (0.62)	0.25
CRP	0.58 (1.30)	1.06 (2.93)	0.98 (2.74)	0.29
AST	23.5 (9.3)	25.4 (14.0)	25.1 (13.4)	0.39
ALT	18.1 (8.9)	21.3 (14.9)	20.8 (14.2)	0.19
HbA1c^‡^	6.33 (1.66)	6.27 (1.18)	6.28 (1.29)	0.81

^*^p-value: chi-square test for categorical variables and *t*-test for continuous variables. Values in bold indicate a *p*-value less than 0.05. ^†^Among patients who survived until discharge. ^‡^Pairwise deletion for missing. NIHSS, National Institute of Health Stroke Scale; mRS, modified Rankin scale; t-PA, tissue plasminogen activator; SBP, systolic blood pressure; DBP, diastolic blood pressure; CRP, C-reactive protein; AST, aspartate aminotransferasel; ALT, alanine aminotransferase.

Procedural characteristics and outcomes are presented in Table 2. The primary occlusion site was not different between the groups (p’s > 0.05 for CCA-ICA, ICA terminus, M1, M2, ACA/PCA, and VBA). Onset-to-door time and puncture-to-recanalization time were comparable between the groups. However, evolocumab users had a slightly longer median door-to-puncture time (145 min [125.5–205.25] vs. 134 min [105.5–168.5], *p* = 0.02) and a higher usage rate of stent retriever (70.7% vs. 53.4%, *p* = 0.04). Aspiration catheter use and angioplasty and/or stenting rates were similar between the groups. The rate of achieving a mTICI of ≥2b was similar between the groups (88.1% vs. 86.3%).

**Table 2:** Procedural characteristics and clinical outcomes between evolocumab user and non-user groups.

	Evolocumab user (*N* = 42)	Non-user (*N* = 219)	Total (*N* = 261)	*p*-value^*^
Median onset-to-door time (IQR)	69 (39.25, 171.25)	60 (31.5, 127)	60 (33, 136)	0.36
Median door-to-puncture time (IQR)	145 (125.5, 205.25)	134 (105.5, 168.5)	135 (109, 176)	**0.02**
Median puncture-to-recanalization time (IQR)	44 (29.5, 59.5)	34 (22, 54)	37 (23, 56)	0.24
**Primary occlusion site** ^†^				
Right side	23 (62.2%)	94 (49.0%)	117 (51.1%)	0.15
CCA-ICA	5 (11.9%)	18 (8.2%)	23 (8.7%)	0.39
ICA terminus	4 (9.5%)	34 (15.5%)	38 (14.4%)	0.47
M1	23 (54.8%)	123 (56.2%)	146 (55.9%)	0.87
M2	4 (9.5%)	14 (6.4%)	18 (6.9%)	0.50
ACA/PCA	1 (2.4%)	5 (2.3%)	6 (2.3%)	1.00
VBA	5 (11.9%)	25 (11.4%)	30 (11.5%)	1.00
**Device used** ^**‡**^ **and procedural result**				
Stent retriever use	29 (70.7%)	117 (53.4%)	147 (56.3%)	**0.04**
Aspiration catheter use	31 (75.6%)	143 (65.3%)	175 (67.0%)	0.21
Angioplasty and/or stenting	4 (9.8%)	19 (8.7%)	23 (8.8%)	0.77
mTICI ≥2b	37 (88.1%)	189 (86.3%)	226 (86.7%)	1.00
**Clinical outcomes**				
Early neurological deterioration	5 (11.9%)	44 (20.1%)	49 (18.8%)	0.21
Any hemorrhage	7 (16.7%)	74 (33.8%)	81 (31.0%)	**0.03**
Symptomatic ICH	1 (2.4%)	19 (8.7%)	20 (7.7%)	0.16
Discharge NIHSS, mean (SD)	8.8 (6.8)	12.4 (9.8)	11.85 (9.49)	**0.02**
Median (IQR)	8 (4, 12)	10 (5, 17)	9 (4, 16)	
Discharge mRS ≤ 3	22 (52.4%)	78 (35.6%)	100 (38.2%)	**0.041**
In hospital death	1 (2.4%)	27 (12.3%)	28 (10.7%)	0.06
3-month mRS ≤ 2^§^	22 (55.0%)	80 (39.2%)	102 (41.8%)	0.06
Death within 3-month^§^	3 (7.5%)	39 (19.1%)	42 (17.2%)	0.08
Stroke recurrence within 3-month^§^	5 (12.5%)	21 (10.4%)	26 (10.8%)	0.70

^*^p-value: chi-square test for categorical variables, *t*-test for continuous variables and Wilcoxon rank-sum test for mRS. Values in bold indicate a *p*-value less than 0.05. ^†^For tandem or multiple occlusions, the main culprit lesion is indicated. “Right side” was checked for occlusion locations within anterior circulation. ^‡^The proportion of devices used during procedures, allowed duplicate checks. ^§^Pairwise deletion for missing. CCA, common carotid artery; ICA, internal carotid artery; M1/M2, 1st and 2nd segments of middle cerebral artery; ACA, anterior cerebral artery; PCA, posterior cerebral artery; mTICI, modified Thrombolysis In Cerebral Infarction; ICH, intercerebral hemorrhage; NIHSS, National Institute of Health Stroke Scale; mRS, modified Rankin scale.

**Figure 1:**
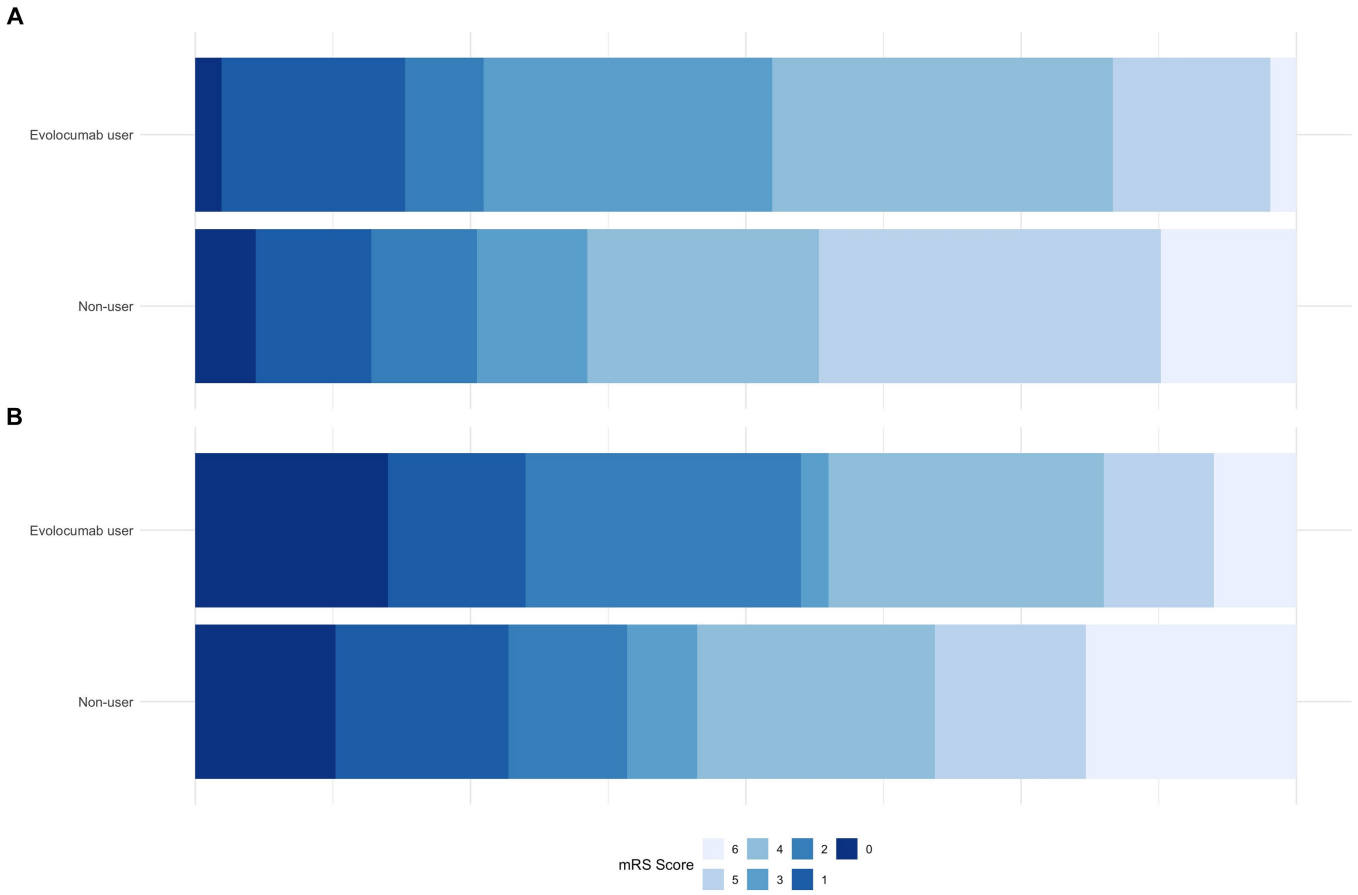
Comparison of mRS proportions between evolocumab user and non-user groups at discharge **(A)** and at 3-month **(B)**.

Notably, evolocumab users had better discharge outcomes, with lower mean discharge NIHSS (8.8 ± 6.8 vs. 12.4 ± 9.8, *p* = 0.02) and a higher proportion of patients with discharge mRS of 0–3 (52.4% vs. 35.6%, *p* = 0.041, Table 2; Figure 1A). Furthermore, incidence of any hemorrhage were significantly lower in evolocumab users (16.7% vs. 33.8%, *p* = 0.03). Although not statistically significant, the incidences of END, sICH, and in-hospital death were also lower in evolocumab users compared to non-users (11.9% vs. 20.1%, *p* = 0.21, 2.4% vs. 8.7%, *p* = 0.16, and 2.4% vs. 12.3%, *p* = 0.06, respectively).

At the 3-month, functional state was assessed in 95.2% of evolocumab users and 93.2% of non-users. Evolocumab users showed a trend toward better outcomes with a higher proportion of patients achieving a mRS of 0–2 and lower mortality (55.0% vs. 39.2, and 7.5% vs. 19.1%, respectively) (Figure 1B); however, the differences were statistically insignificant (*p* = 0.06 and 0.08, respectively). Stroke recurrence rates at 3 months were similar between the groups.

### Predictor of favorable functional outcome

In univariate analysis, sex, age, initial NIHSS score, cardioembolic stroke subtype, hypertension, diabetes, and smoking were associated with discharge mRS of 0–3 (Table 3). Use of evolocumab was associated with a favorable discharge functional outcome (*p*-value = 0.043). Besides, stent retriever use and pre-stroke statin use, which differed in proportion between evolocumab users and nonusers, were not associated with a discharge mRS of 0–3.

**Table 3:** Multivariable analysis for predictors of discharge mRS ≤ 3.

			Model 1^*^		Model 2^†^		Model 3^‡^	
Variable	Crude OR (95% CI)	*p*-value	Adjusted OR (95% CI)	*p*-value	Adjusted OR (95% CI)	*p*-value	Adjusted OR (95% CI)	*p*-value
Evolocumab	1.99 (1.02–3.89)	**0.043**	2.10 (1.03–4.33)	**0.043**	2.02 (0.97–4.24)	0.06	1.98 (0.94–4.22)	0.07
Men	2.10 (1.25–3.56)	**<0.01**	1.93 (1.11–3.39)	**0.02**	1.56 (0.86–2.86)	0.15	1.58 (0.87–2.91)	0.14
Age	0.97 (0.95–0.99)	**0.01**	0.97 (0.95–1.00)	**0.02**	0.99 (0.96–1.02)	0.49	0.99 (0.96–1.02)	0.49
Iinial NIHSS	0.90 (0.86–0.94)	**<0.001**	0.90 (0.86–0.94)	**<0.001**	0.90 (0.86–0.94)	**<0.001**	0.90 (0.86–0.94)	**<0.001**
Cardioembolism	0.52 (0.31–0.87)	**0.01**			0.93 (0.51–1.71)	0.81	0.91 (0.49–1.68)	0.76
Hypertension	0.47 (0.28–0.78)	**<0.01**			0.64 (0.35–1.17)	0.14	0.62 (0.33–1.15)	0.13
Diabetes	0.59 (0.32–1.04)	0.07			0.61 (0.31–1.17)	0.14	0.60 (0.31–1.15)	0.13
Smoking	2.91 (1.66–5.15)	**<0.001**			2.06 (1.03–4.14)	**0.04**	2.08 (1.04–4.20)	**0.04**
Stent retriever use	0.98 (0.59–1.62)	0.93					1.20 (0.68–2.14)	0.54
Pre-stroke statin	0.90 (0.50–1.62)	0.73					1.15 (0.58–2.25)	0.69

Values in bold indicate a *p*-value less than 0.05. ^*^Model 1 adjusted for demographics and initial NIHSS. ^†^Model 2 further adjusted for variables with *p* < 0.01 in the univariate analyses. ^‡^Model 3 additionally adjusted for variables that differed between groups.

In the simplest model (Model 1; adjust only demographics and initial NIHSS), evolocumab use was an independent predictor for a discharge mRS of 0–3 (adjusted OR 2.10, 95% CI 1.03–4.33, *p* = 0.043). However, its significance was reduced upon adjusting for additional variables. In Models 2 and Model 3, the association between evolocumab use and discharge mRS of 0–3 was marginally significant (adjusted OR 2.02, 95% CI 0.97–4.24, *p* = 0.06 and adjusted OR 1.98, 95% CI 0.94–4.22, *p* = 0.07, respectively).

**Figure 2:**
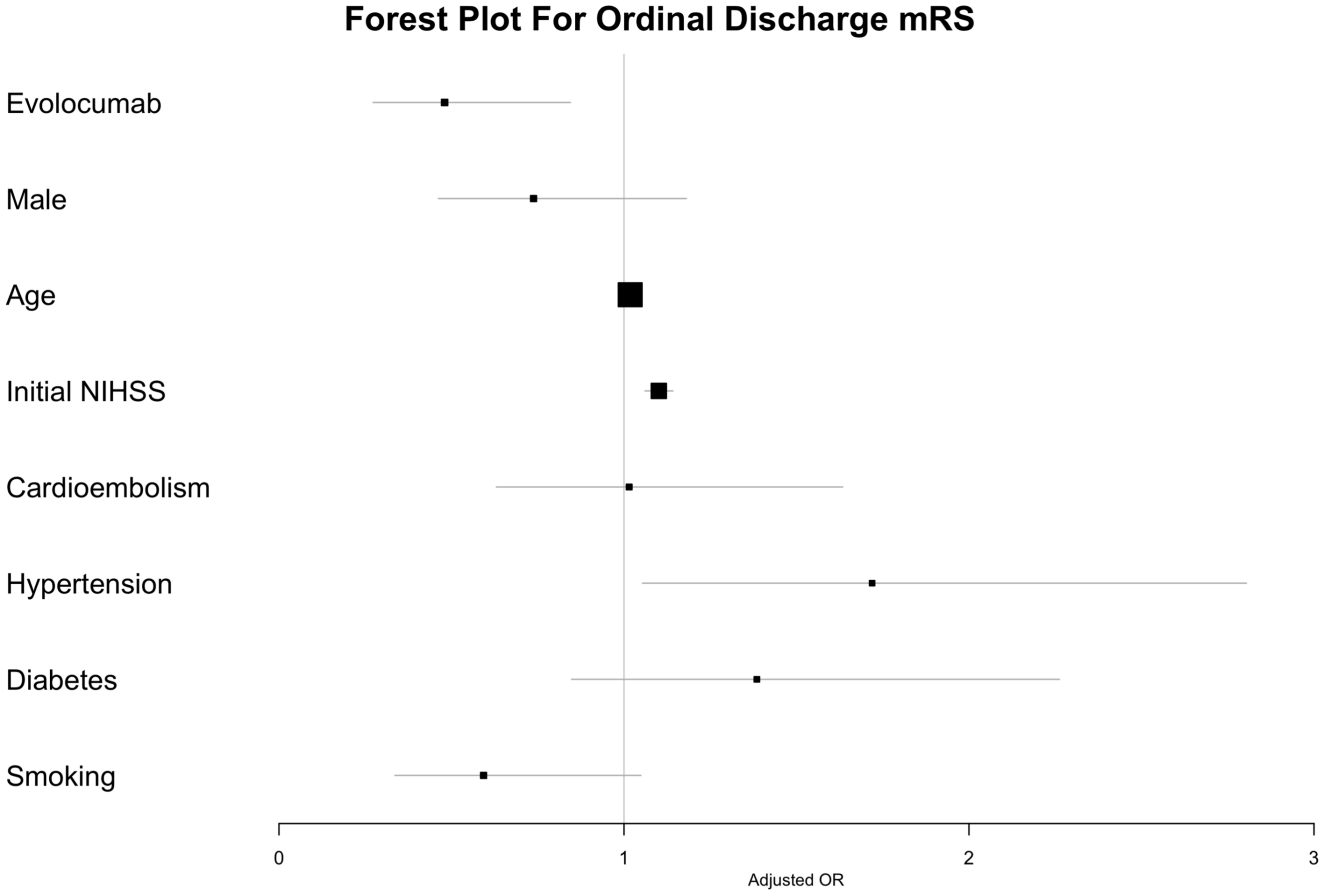
Predictors of lower discharge mRS as ordinal scale.

In the multivariate ordinal logistic regression assessing the impact of evolcumab use on discharge mRS, a statistically significant association was observed with a reduction in discharge mRS scores. The significance persisted across multiple models: the simplest Model 1; Model 2, which adjusted for variable associated with discharge mRS; and Model 3, which accounted for differences between the evolocumab user and nonuser groups (Table 4; Figure 2). Additionally, significant associations were noted with the initial NIHSS score and the presence of hypertension. In the multivariate ordinal logistic regression for 3-month mRS, evolocumab use exhibited a marginal association (*p* = 0.09, 0.08, and 0.09 for Models 1, 2, and 3, Supplementary Table S1; Supplementary Figure S2). However, significant associations were found for age, initial NIHSS score, and hypertension with 3-month mRS.

**Table 4:** Multivariable analysis for ordinal discharge mRS score.

			Model 1		Model 2		Model 3	
Variable	Crude OR (95% CI)	*p*-value	Adjusted OR (95% CI)	*p*-value	Adjusted OR (95% CI)	*p*-value	Adjusted OR (95% CI)	*p*-value
Evolocumab	0.52 (0.30–0.89)	**0.02**	0.47 (0.27–0.83)	**0.01**	0.48 (0.27–0.85)	**0.01**	0.47 (0.27–0.84)	**0.01**
Men	0.57 (0.37–0.88)	**0.01**	0.64 (0.41–0.99)	**0.047**	0.74 (0.46–1.18)	0.21	0.72 (0.45–1.16)	0.18
Age	1.03 (1.01–1.05)	**<0.01**	1.03 (1.01–1.05)	**<0.01**	1.02 (1.00–1.04)	0.10	1.02 (1.00–1.04)	0.12
Iinial NIHSS	1.10 (1.06–1.14)	**<0.001**	1.10 (1.06–0.94)	**<0.001**	1.10 (1.06–1.14)	**<0.001**	1.10 (1.06–1.14)	**<0.001**
Cardioembolism	1.78 (1.15–2.76)	**<0.0**			1.02 (0.63–1.63)	0.95	1.04 (0.64–1.67)	0.89
Hypertension	2.33 (1.48–3.69)	**<0.001**			1.72 (1.05–2.80)	**0.03**	1.84 (1.11–3.04)	**0.02**
Diabetes	1.54 (0.96–2.48)	0.07			1.39 (0.85–2.26)	0.19	1.44 (0.88–2.37)	0.15
Smoking	0.38 (0.23–0.62)	**<0.001**			0.59 (0.34–1.05)	0.07	0.60 (0.34–1.05)	0.08
Stent retriever use	1.04 (0.67–1.60)	0.87					0.92 (0.59–1.44)	0.71
Pre-stroke statin	0.99 (0.61–1.63)	0.98					0.74 (0.44–1.26)	0.27

Values in bold indicate a *p*-value less than 0.05. ^*^Model 1 adjusted for demographics and initial NIHSS. ^†^Model 2 further adjusted for variables with *p* < 0.01 in the univariate analyses. ^‡^Model 3 additionally adjusted for variables that differed between groups.

### Lipid profile

In the baseline lipid panel, evolocumab users had slightly higher total cholesterol levels compared to non-users, although this difference was not statistically significant (178.9 vs. 163.5, *p* = 0.07, Supplementary Table S2). The levels of LDL, TG, and HDL were also similar between the two groups (p’s = 0.39, 0.30, and 0.30, respectively).

At 3-month, 64.1% of evolocumab users and only 31.7% of non-users had completed the lipid panel follow-up. No significant differences were observed between the two groups in 3-month total cholesterol, LDL, and HDL levels. However, the interval changes in lipid levels from baseline to follow-up showed greater reductions in total cholesterol (−58.9 vs. -45.3, *p* = 0.12) and LDL (−48.1 vs. -28.1, *p* = 0.01) in evolocumab users compared to non-users.

Patients who had a 3-month lipid follow-up demonstrated significantly higher statin prescriptions at discharge than 3-month stroke survivors without lipid follow-up. However, there was no difference in other characteristics, including initial stroke severity, discharge NIHSS and discharge mRS (data not shown). Although these findings in lipid profile were based on a small subset of patients, they confirmed a potential benefit of evolocumab in reducing LDL-cholesterol levels over time.

## Discussion

In this study, the early use of evolocumab was associated with lower discharge NIHSS and a higher proportion of patients achieving lower mRS scores, suggesting improved functional outcomes. However, statistical significance was not maintained at 3-month follow-up. Patients who treated with early evolocumab experienced fewer instances of END, sICH, and in-hospital deaths, along with a significant reduction in LDL-cholesterol levels.

To our knowledge, this is the first study to explore the early use of evolocumab in AIS patients underwent MT without specific indications. Our cohort demonstrated a discharge mRS 0–3 proportion of 38.2% and a 3-month mRS 0–2 proportion of 41.8%. We defined a discharge mRS of 0–3, not a traditional mRS of 0–2, as an indicator for a *‘favorable functional outcome’*, considering the potential for enhanced benefits of rehabilitation in patients receiving reperfusion therapy. Interestingly, the discharge mRS 0–3 rates closely matched the 3-month mRS 0–2 rates. However, these were slightly lower that 46% rate reported in a meta-analysis of major MT trials (21). This discrepancy may be due to the inclusion of 7.7% of our patients with a pre-stroke mRS of two, whereas most studies in the HERMES collaboration included patients with pre-stroke mRS 0–1 (21–23).

Evolocumab users had a 1.4-fold higher proportion of discharge mRS 0–3 than non-users (*p* = 0.043). After adjusting for demographics, initial NIHSS scores, and other potential contributing factors such as hypertension, diabetes, current smoking, stroke subtype, stent retriever use, and pre-stroke statin use, the statistical significance diminished. However, the association between evolocumab and a lower discharge mRS remained significant in the multivariate ordinal logistic regression model, even after adjusting for the variables (p’s = 0.01 for Model 1, 2 and 3).

We proposed two mechanisms for the beneficial effects of early PCSK9 inhibitor use during MT: (1) The inhibition of PCSK9 could potentially prevent vessel re-occlusion and reduce infarct size. This hypothesis is supported by findings from a pilot randomized trial that indicated a trend toward an increased myocardial salvage index with immediate administration of PCSK9 inhibitor following coronary intervention (15); (2) PCSK9 inhibitors might reduce the hemorrhagic transformation in the reperfused area, thereby leading to favorable functional outcomes, as evidenced by the lower incidence of sICH in evolocumab users in our study.

While pivotal trials spotlighted the LDL-lowering capabilities of PCSK9 inhibitors, recent studies have focused on the pleiotropic effects of PCSK9 inhibitors on atherosclerosis beyond LDL reduction (24). PCSK9 modulates inflammation, atherosclerosis progression, platelet activation, and thrombus formation. In animal models, application of alirocumab and the anti-PCSK9 AT04A vaccine have been shown to reduce vascular inflammation and atherosclerotic lesions (12, 25). There are several recent or ongoing studies examining the early use of PCSK9 inhibitors in acute coronary syndrome patients undergoing percutaneous coronary intervention with anticipated extra-LDL reduction and other pleiotropic effects (15, 26, 27).

The addition of PCSK9 inhibitors to high-dose statin has been shown to reduce the occurrence of stroke, MI, and cardiovascular death in patients with vascular diseases or high-risk features (5, 6). The greater LDL reduction with PCSK9 inhibitors has been associated with fewer cardiovascular events. In our study, evolocumab users exhibited a significantly greater LDL reduction at 3 months than non-users (mean −48.1 mmol/L vs. −28.1 mmol/L, *p* = 0.01). However, given the retrospective nature of our study and incomplete lipid panel follow-ups, further systematic study is necessary to clarify the relationship between LDL reduction and functional outcomes after AIS.

Few epidemiologic studies and the SPARCL study have linked LDL lowering with intracerebral hemorrhage (28, 29), raising concerns that excessive LDL lowering could increase the risk for hemorrhage. However, our study did not find an increase in sICH or any hemorrhagic transformation in evolocumab users compared to non-users. This is consistent with the FOURIER study (5), which reported no significant increase in intracerebral hemorrhage, and meta-analyses that showed no increase in hemorrhage even in subgroup with extremely low LDL levels (30). Instead, the improving functional outcome may be attributed to a significant reduction in hemorrhage in the post-procedural period.

Our study has several limitations. First, the small number of patients treated with evolocumab at our center constrains the statistical power, resulting in only a trend toward significance after adjustment. Moreover, the smaller subset of patients available for the 3-month follow-up precludes the demonstration of medium-term effects of the treatment. To overcome this limitation, we plan to conduct a subsequent multicenter study. Second, the non-randomized, retrospective observational design may introduce selective bias and prevent the establishment of a causal relationship between evolocumab use and outcomes. Nonetheless, aside from differences in stent retriever use and pre-stroke statin use, there were no significant differences in the demographics, vascular risk factors, and mTICI grade between evolocumab-users and non-users. Third, due to national insurance policies and cost considerations, evolocumab was administered only once, and follow-up on LDL levels was not comprehensive. As a result, it was difficult to fully assess the LDL-lowering effect of evolocumab in our cohort.

In conclusion, our study suggests that PCSK9 inhibitors could potentially benefit discharge outcomes for AIS patients who undergo MT. PCSK9 inhibitors may contribute to favorable outcomes by reducing the incidence of hemorrhage and END. However, these findings, derived from a small cohort, need validation in larger, prospective studies.

## Data availability statement

The raw data supporting the conclusions of this article will be made available by the authors, without undue reservation.

## Ethics statement

The studies involving humans were approved by Inha University Hospital Institutional Review Border. The studies were conducted in accordance with the local legislation and institutional requirements. The ethics committee/institutional review board waived the requirement of written informed consent for participation from the participants or the participants’ legal guardians/next of kin because retrospective study design and the minimal risk posed to the participants.

## Author contributions

JK: Conceptualization, Data curation, Formal analysis, Investigation, Methodology, Resources, Validation, Visualization, Writing – original draft, Writing – review & editing. UH: Data curation, Formal analysis, Resources, Validation, Visualization, Writing – review & editing. CY: Data curation, Investigation, Methodology, Resources, Validation, Writing – review & editing. JB: Data curation, Investigation, Methodology, Resources, Validation, Writing – review & editing. J-HR: Conceptualization, Investigation, Methodology, Resources, Supervision, Validation, Writing – review & editing. H-KP: Conceptualization, Investigation, Methodology, Project administration, Resources, Supervision, Validation, Writing – review & editing.

## Footnotes

^1^ http://www.r-project.org/
