# Supplementary material for: PCSK9 inhibitor in acute ischemic stroke patient receiving mechanical thrombectomy: early outcomes and safety
Source: Front Neurol. 2024 May 16;15:1375609. doi: 10.3389/fneur.2024.1375609 (PMC11137246; doi:10.3389/fneur.2024.1375609)

**PCSK9 Inhibitor in Acute Ischemic Stroke Patient Receiving Mechanical Thrombectomy: Early Outcomes and Safety**

**SUPPLEMENTAL MATERIAL**

Table S1. Multivariable Analysis for Ordinal 3-month mRS Score.

Table S2. Lipid Profile Between Evolocumab User and Non-user Groups.

Table S3. Comparison Between the Patient With LDL Follow-up and Without LDL Follow-up (among 3-month survivor).

Figure S1. Flow Chart of Study Population Selection

Figure S2. Predictors of Lower 3-month mRS as Ordinal Scale.

**Table S1.** Multivariable Analysis for Ordinal 3-month mRS Score.

|  |  |  | Model 1 | | Model 2 | | Model 3 | |
| --- | --- | --- | --- | --- | --- | --- | --- | --- |
| Variable | Crude OR (95% CI) | p-value | Adjusted OR  (95% CI) | p-value | Adjusted OR  (95% CI) | p-value | Adjusted OR  (95% CI) | p-value |
| Evolocumab | 0.61 (0.34 – 1.08) | 0.09 | 0.70 (0.33 – 1.08) | 0.09 | 0.58 (0.32 – 1.06) | 0.08 | 0.59 (0.32 – 1.09) | 0.09 |
| Men | 0.61 (0.39 – 0.95) | **0.03** | 0.72 (0.46 – 1.14) | 0.16 | 0.80 (0.49 – 1.31) | 0.38 | 0.78 (0.48 – 1.28) | 0.33 |
| Age | 1.03 (1.02 – 1.05) | **<.001** | 1.03 (1.02 – 1.05) | **<.001** | 1.02 (1.00 – 1.04) | 0.049 | 1.02 (1.00 – 1.04) | 0.054 |
| Iinial NIHSS | 1.10 (1.06 – 1.14) | **<.001** | 1.10 (1.06 – 1.14) | **<.001** | 1.09 (1.05 – 1.14) | <.001 | 1.09 (1.05 – 1.14) | <.001 |
| Cardioembolism | 1.70 (1.09 – 2.66) | **0.02** |  |  | 0.91 (0.56 – 1.50) | 0.72 | 0.96 (0.58 – 1.59) | 0.88 |
| Hypertension | 2.65 (1.66 – 4.25) | **<.001** |  |  | 1.93 (1.15 – 3.22) | 0.01 | 2.11 (1.25 – 3.56) | <0.01 |
| Diabetes | 1.70 (1.04 – 2.79) | **0.03** |  |  | 1.38 (0.82 – 2.31) | 0.23 | 1.41 (0.84 – 2.37) | 0.20 |
| Smoking | 0.44 (0.27 – 0.73) | **<0.01** |  |  | 0.70 (0.39 – 1.26) | 0.24 | 0.71 (0.40 – 1.27) | 0.25 |
| Stent retriever use | 0.82 (0.52 – 1.28) | 0.38 |  |  |  |  | 0.69 (0.44 – 1.10) | 0.12 |
| Pre-stroke statin | 0.88 (0.54 – 1.44) | 0.61 |  |  |  |  | 0.75 (0.45 – 1.26) | 0.28 |

* Model 1 adjusted for demographics and initial stroke severity only.

† Model 2 further adjusted for variables that were associated in the univariate analysis.

‡ Model 3 additionally adjusted for variables that differed between evolocumab users and non-users

**Table S2. Lipid Profile Between Evolocumab User and Non-user Groups.**

|  | **Evolocumab user** | **Non-user** | **Total** | **p value*** |
| --- | --- | --- | --- | --- |
| **Baseline lipid panel** | | | | |
| Total cholesterol | 176.9 (46.9) | 163.5 (42.2) | 165.6 (43.2) | 0.07 |
| LDL | 100.9 (39.6) | 95.1 (38.0) | 96.12 (38.3) | 0.39 |
| HDL | 44.7 (12.2) | 42.5 (12.3) | 42.84 (12.3) | 0.30 |
| TG | 107.4 (62.2) | 119.9 (71.3) | 117.7 (69.9) | 0.30 |
| **Follow-up lipid panel**† | | | | |
| F/U Total cholesterol | 118.6 (37.5) | 116.7 (34.6) | 117.1 (35.1) | 0.77 |
| F/U LDL | 61.6 (31.6) | 63.7 (25.2) | 63.0 (27.1) | 0.73 |
| F/U HDL | 46.4 (12.1) | 43.2 (14.4) | 44.2 (13.7) | 0.29 |
| F/U TG | 90.8 (33.4) | 112.0 (54.5) | 105.7 (50.0) | 0.051 |
| **Interval changes**† | | | | |
| Total cholesterol | -58.9 (51.6) | -45.3 (47.4) | -48.0 (48.4) | 0.12 |
| LDL | -48.1 (34.8) | -28.1 (34.6) | -34.4 (35.8) | **0.01** |
| HDL | +1.7 (11.3) | +1.7 (11.7) | +1.7 (11.5) | 0.99 |
| TG | -15.1 (45.0) | -8.3 (83.7) | -10.4 (73.5) | 0.68 |

* p-value by t-test between groups.

† Pairwise deletion for missing.

LDL, low density lipoprotein; HDL, high density lipoprotein; TG, triglyceride

**Table S3.** Comparison Between the Patient With LDL Follow-up and Without LDL Follow-up (among 3-month survivor).

|  | LDL follow-up group (N=96) | No Follow-up group (N=106) | Total (N=202) | p value |
| --- | --- | --- | --- | --- |
| Male | 54 (56.2%) | 65 (61.3%) | 119 (58.9%) | 0.464 |
| Age, mean (SD) | 67.19 (11.82) | 68.57 (12.40) | 67.91 (12.11) | 0.421 |
| Pre-stroke mRS |  |  |  |  |
| 0 | 83 (86.5%) | 95 (89.6%) | 178 (88.1%) |  |
| 1 | 6 (6.2%) | 4 (3.8%) | 10 (5.0%) |  |
| 2 | 7 (7.3%) | 7 (6.6%) | 14 (6.9%) |  |
| Initial NIHSS, median (IQR) | 14.5 (11.75, 18) | 14.5 (10, 18) | 14.5 (11, 18) | 0.343 |
| - Mean (SD) | 15.31 (6.38) | 15.46 (6.33) | 15.41 (6.34) |  |
| **Risk factor** | | | | |
| Hypertension | 16 (16.7%) | 13 (12.3%) | 29 (14.4%) | 0.373 |
| Diabetes | 56 (58.3%) | 59 (55.7%) | 115 (56.9%) | 0.702 |
| Dyslipidemia | 28 (29.2%) | 25 (23.6%) | 53 (26.2%) | 0.368 |
| Atrial fibrillation | 23 (24.0%) | 36 (34.0%) | 59 (29.2%) | 0.118 |
| Past stroke history | 42 (43.8%) | 46 (43.4%) | 88 (43.6%) | 0.96 |
| Current smoker | 27 (28.1%) | 34 (32.1%) | 61 (30.2%) | 0.541 |
| Obesity (BMI≥30) | 5 (5.2%) | 11 (10.4%) | 16 (7.9%) | 0.174 |
| **Stroke Subtype** |  |  |  | 0.594 |
| Large artery atherosclerosis | 23 (24.0%) | 32 (30.2%) | 55 (27.2%) |  |
| Cardioembolism | 42 (43.8%) | 44 (41.5%) | 86 (42.6%) |  |
| Others | 31 (32.3%) | 30 (28.3%) | 61 (30.2%) |  |
| rTPA | 35 (36.5%) | 32 (30.2%) | 67 (33.2%) | 0.345 |
| Prior statin use | 25 (26.0%) | 28 (26.4%) | 53 (26.2%) | 0.952 |
| **Discharge statin use** | **90 (93.8%)** | **88 (83.0%)** | **178 (88.1%)** | 0.019 |
| Discharge antiplatelet use | 50 (52.1%) | 65 (61.3%) | 115 (56.9%) | 0.185 |
| Discharge anticoagulant use | 44 (45.8%) | 41 (38.7%) | 85 (42.1%) | 0.304 |
| SBP, mean (SD) | 152.18 (24.98) | 153.37 (31.19) | 152.80 (28.34) | 0.766 |
| DBP, mean (SD) | 85.52 (16.76) | 88.14 (20.29) | 86.90 (18.70) | 0.321 |
| Cr, mean (SD) | 0.91 (0.72) | 0.91 (0.26) | 0.91 (0.53) | 0.978 |
| Hb A1c, mean (SD) | 6.19 (1.07) | 6.33 (1.37) | 6.25 (1.21) | 0.5 |
| LDL, mean (SD) | 98.80 (36.95) | 95.51 (36.67) | 97.09 (36.74) | 0.545 |
| HDL, mean (SD) | 44.11 (14.22) | 42.64 (11.09) | 43.35 (12.68) | 0.43 |
| Tg, mean (SD) | 113.38 (76.52) | 119.06 (70.11) | 116.33 (73.12) | 0.599 |
| **Procedure time matrix** | | | | |
| Median onset-to-door time (IQR) | 59.50 (33.00, 134.50) | 61.00 (40.50, 151.75) | 60.00 (35.25, 141.00) | 0.286 |
| Median door-to-puncture time (IQR) | 128.00 (103.50, 162.50) | 133.50 (111.00, 175.25) | 133.00 (109.00, 171.75) | 0.855 |
| Median puncture-to-recanalization time (IQR) | 32.00 (22.00, 50.00) | 37.00 (24.25, 56.00) | 34.00 (23.00, 52.00) | 0.325 |
| **Occlusion Location** | | | | |
| Right side | 43 (50.6%) | 46 (47.9%) | 89 (49.2%) | 0.72 |
| CCA-ICA | 7 (7.3%) | 11 (10.4%) | 18 (8.9%) | 0.442 |
| ICA terminus | 8 (8.3%) | 19 (17.9%) | 27 (13.4%) | 0.045 |
| M1 | 59 (61.5%) | 56 (52.8%) | 115 (56.9%) | 0.216 |
| M2 | 10 (10.4%) | 6 (5.7%) | 16 (7.9%) | 0.211 |
| ACA/PCA | 1 (1.0%) | 5 (4.7%) | 6 (3.0%) | 0.124 |
| VBA | 11 (11.5%) | 9 (8.5%) | 20 (9.9%) | 0.481 |
| mTICI ≥2b | 88 (91.7%) | 90 (84.9%) | 178 (88.1%) | 0.138 |
| **Clinical Outcome** | | | | |
| Discharge NIHSS, mean (SD) | 8.79 (6.03) | 9.00 (6.52) | 8.90 (6.28) | 0.815 |
| Discharge mRS ≤3 | 43 (44.8%) | 47 (44.3%) | 90 (44.6%) | 0.949 |
| 3-month mRS ≤2 | 52 (54.2%) | 50 (47.2%) | 102 (50.5%) | 0.321 |

**Figure S1**. Flow Chart of Study Population Selection


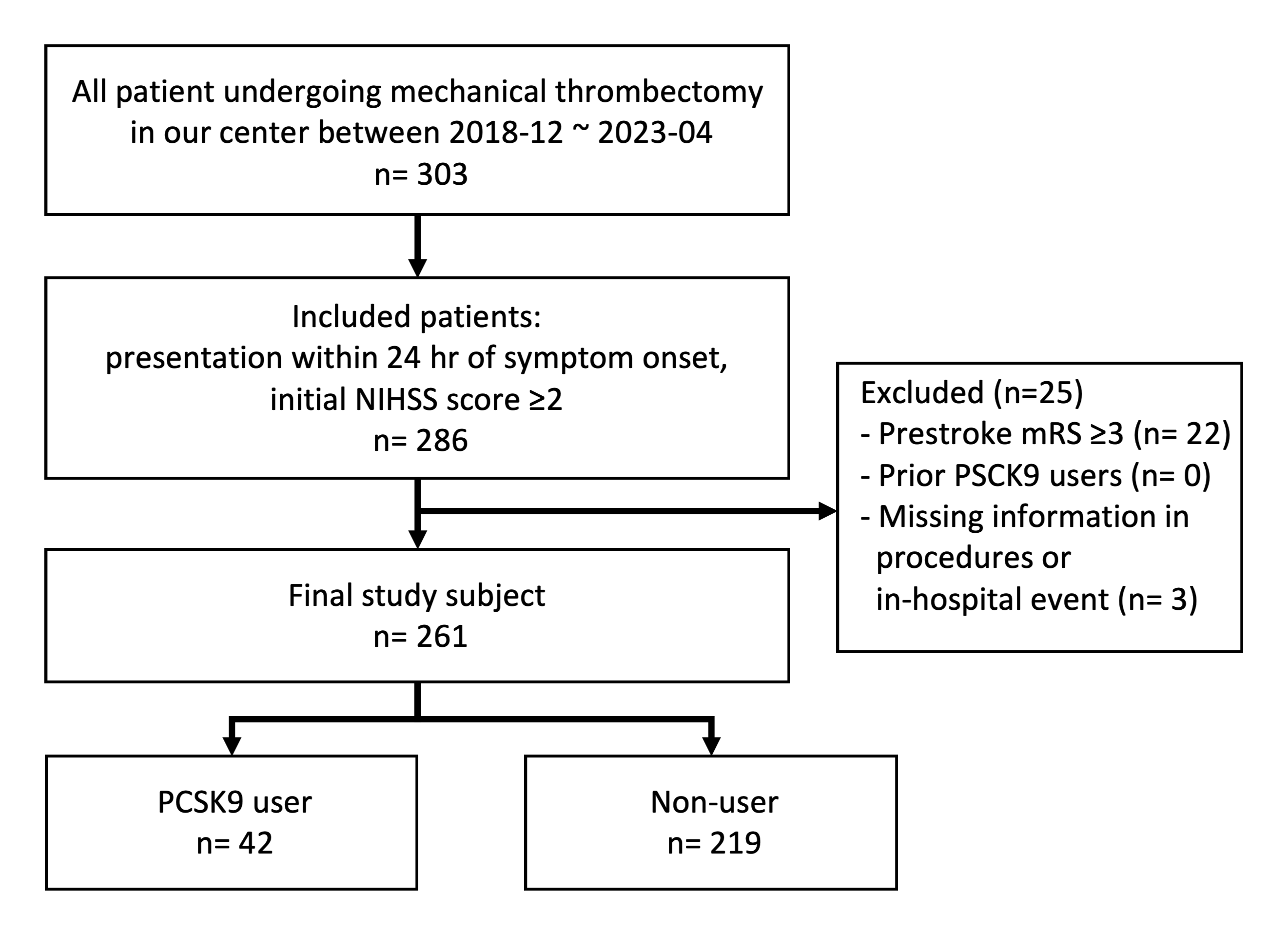


**Figure S2**. Predictors of Lower 3-month mRS as Ordinal Scale.


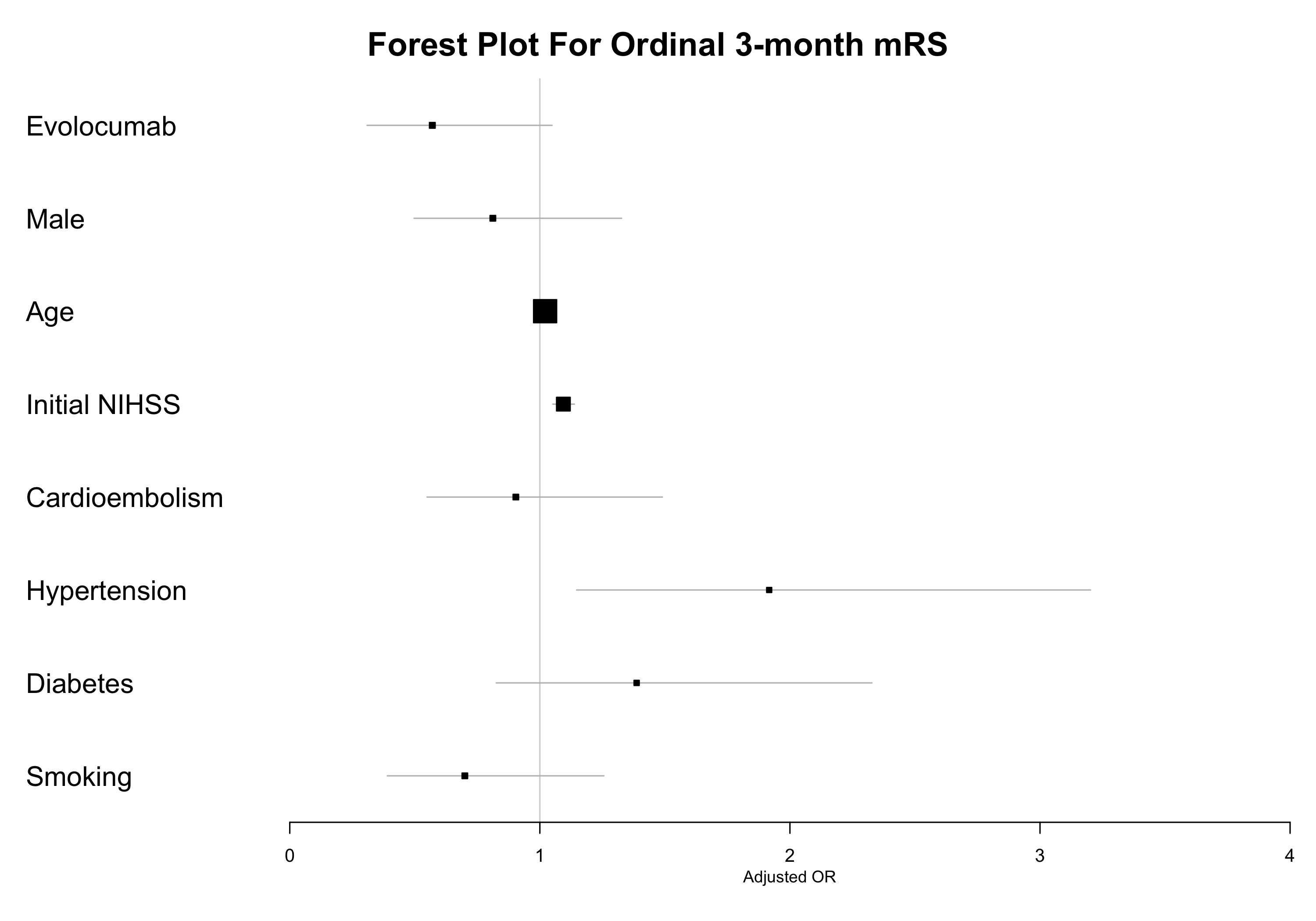

Supplement: Supplementary file 1 [file Data_Sheet_1.docx]
